# Mammalian conserved ADAR targets comprise only a small fragment of the human editosome

**DOI:** 10.1186/gb-2014-15-1-r5

**Published:** 2014-01-07

**Authors:** Yishay Pinto, Haim Y Cohen, Erez Y Levanon

**Affiliations:** 1 Mina and Everard Goodman Faculty of Life Sciences, Bar-Ilan University, Ramat Gan, Israel

## Abstract

**Background:**

ADAR proteins are among the most extensively studied RNA binding proteins. They bind to their target and deaminate specific adenosines to inosines. ADAR activity is essential, and the editing of a subset of their targets is critical for viability. Recently, a huge number of novel ADAR targets were detected by analyzing next generation sequencing data. Most of these novel editing sites are located in lineage-specific genomic repeats, probably a result of overactivity of editing enzymes, thus masking the functional sites. In this study we aim to identify the set of mammalian conserved ADAR targets.

**Results:**

We used RNA sequencing data from human, mouse, rat, cow, opossum, and platypus to define the conserved mammalian set of ADAR targets. We found that the conserved mammalian editing sites are surprisingly small in number and have unique characteristics that distinguish them from non-conserved ones. The sites that constitute the set have a distinct genomic distribution, tend to be located in genes encoding neurotransmitter receptors or other synapse related proteins, and have higher editing and expression levels. We also found a high consistency of editing levels of this set within mice strains and between human and mouse. Tight regulation of editing in these sites across strains and species implies their functional importance.

**Conclusions:**

Despite the discovery of numerous editing targets, only a small number of them are conserved within mammalian evolution. These sites are extremely highly conserved and exhibit unique features, such as tight regulation, and probably play a pivotal role in mammalian biology.

## Background

The canonical post-transcriptional modification of adenosine to inosine (A-to-I) deamination is catalyzed by enzymes of the ADAR family, which bind to double-stranded RNA (dsRNA) [1-3]. This reaction takes place in a wide variety of organisms of the metazoan lineage [4-9]. A-to-I substitution causes the intracellular translation machinery to identify inosine (I) as guanosine (G), and thus, can lead to protein diversification. In addition to creating synonymous and non-synonymous codon changes, several additional functional and regulatory implications were also found for editing. RNA editing is involved in several processes, including: alteration of pre-mRNA splicing by the creation or elimination of splice sites [10-12]; RNA degradation [13,14]; viral RNA replication [15]; nuclear retention of transcripts [16]; miRNA regulation [17,18]; and protein susceptibility to proteolytic cleavage [19].

Vast amounts of sequencing data have become available over the last few years. Consecutive computational approaches were developed to identify novel RNA editing sites, taking advantage of the available large RNA sequencing (RNA-seq) datasets. Such analysis is generally performed by comparing DNA to RNA sequences [12,20-24]. Editing events are detected as A-to-G mismatches between the reference genome and the RNA reads. Although the concept is simple, these approaches are very susceptible to false positives due to a low signal-to-noise ratio, caused by sequencing and alignment errors and mismatches derived from somatic mutations and polymorphisms in the population [22,25-27]. The current rapid progress in sequencing technologies, led to the publication of a huge number of editing sites, more than a million in human [20,28], and thousands of additional ones in mouse [4,29] and *Drosophila*[5,30]. Most of these sites are consequences of double-stranded RNA structures formed by inverted, usually lineage specific, repeats (for example, *Alu* pairs [12,31-33] in human, and B1 in mouse [29]). Yet, it is not clear which of the sites have functional importance, and how many are only the outcomes of residual ADAR activity, with no selective advantage.

Although most of the sites are located in genomic repeats, and seem to have no functional importance, RNA editing is an essential process, as ADAR1^−/−^ and ADAR2^−/−^ mice exhibit embryonic and postnatal lethal phenotypes, respectively [34,35], and editing is involved in several key cellular functions and diseases [35-39]. Indeed, important biological functions were assigned to several recoding sites (editing sites that change the translated protein sequence). Most of these established sites are located in neuronal genes, such as the editing site in the glutamate receptor (GLUR2) gene that leads to a non-synonymous substitution (Q607R), which takes place in glutamatergic neurons in close to 100% of the transcripts (100% editing levels) [40]. Other examples are the cluster of five editing events in the serotonin receptor 5HT_2C_R which regulates mood, appetite, and behavior [41-43], and the editing event in the voltage-gated potassium channel, Kcna1 [44].

In this study, we wished to identify from within the large number of novel editing sites, those sites that became important over the course of mammalian evolution. In order to achieve this goal, we used evolution as the key selection tool, looking for sites that are common in several lineages, which suggest that they were functionally selected. Creating such a catalog of selected editing sites will have a remarkable effect on functional editing research, since it illuminates the few ‘meaningful’ sites, by not only defining the functional sites, but also by suggesting a method to define them.

Surprisingly, we found that the number of such conserved editing sites is extremely small, and probably only a limited set of such functionally important editing sites exist. We found that the plethora of sequencing data did not contribute much to the discovery of novel conserved sites, as most of the functionally important sites were known before the next generation sequencing revolution. Nevertheless, we were able to discover, based on their extreme conservation, the few editing sites that probably play a pivotal role in mammalian biology. In addition, our results demonstrate that editing, in parallel to the established mutational processes that shape genomes, add another layer of variability and complexity that can be spatiotemporally regulated.

## Results

### The conserved editing set is small

Most of the known editing sites seem to be located in lineage-specific regions, mainly in inverted repeats as is the case in the human [20,28], mouse [4], and fruit fly [5]. Only very few sites are known to be conserved across large evolutionary distances. For example, only one site was found to be conserved between human and *Drosophila* fly [45,46] (probably due to convergent evolution) and only a handful of sites were found to be conserved between human and mouse, so far [21]. In the last few years, the total number of known human editing sites jumped by several orders of magnitude; thus, many expected that the number of functional sites would grow at the same rate.

**Figure 1:**
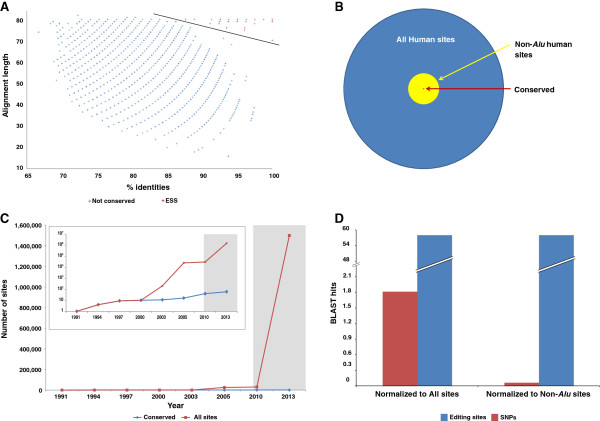
**Mammalian set of editing sites. (A)** BLAST hits for human-mouse editing sets alignment, the Y axis represents the alignment length and the X axis represents the identity percent. The conserved set is colored red, non-conserved hits are colored blue, and the linear separator is colored in black. **(B)** Venn diagram of human editing sites shows that only a tiny fraction of the editing sites are conserved. The conserved sites are small minority of the non-*Alu* sites, as well. All sites (1,432,744) are colored blue, non-*Alu* sites (52,312) are colored yellow, and 59 conserved sites are colored red. **(C)** Number of total known editing sites (red) and conserved (blue) since the identification of the first editing sites, until today. Identification of sites using next generation sequencing technologies began in 2009; this period is colored in gray. While the total number of editing sites increased by six orders of magnitude during this period, the number of conserved sites barely increased. **(D)** Hit enrichment for editing sites compared to SNPs using exactly the same pipeline shows high signal-to-noise ratio. The number of hits was normalized to all sites dataset sizes (left) and to non-Alu sites (right).

In order to build a comprehensive and updated dataset of conserved mammalian editing sites, we collected all available RNA editing sites from recent RNA-seq studies of both human and mouse. This dataset contains a total of 1,432,743 human sites [20,28,47] and 10,210 [4,47-50] mouse sites. All were found by aligning large sets of RNA sequences, in an unbiased manner, to the matched genomes. In order to find sites that are highly conserved between species, we retrieved for each site the 80 bp flanking genomic sequence (40 nucleotides upstream and 40 downstream) and aligned each of the human sequences to all mouse sequences using the standard BLAST [51] alignment tool. We filtered out sites below stringent alignment thresholds (an identity of at least 70 of the 81 nucleotides), and retained only sites in which the A-to-G mismatch appears in both human and mouse at the same position (see Methods). Applying this straightforward procedure resulted in 59 evolutionary selected sites (ESS) (Table 1, Figure 1A-B and in Additional file 1: Table S1). Surprisingly, we found that the number of sites in the ESS is very small (0.004% of human sites) and increased only slightly in recent years, while the total number of sites grow by several orders of magnitude (Figure 1C). We found that this set was hardly affected by changing the alignment parameters. In addition, we obtained similar results when we used the UCSC lift over tool, which converts coordinates between genomes [52] (see Additional file 1: Table S2), suggesting that this set is coherent and unique (only one additional coding target in the GLI gene was added by this method). The sensitivity of this approach appears to be very high as the set contains all the previously well characterized sites. Even though there was a dramatic increase in the number of identified editing sites over the last few years, the number of conserved sites remains low. In order to estimate the selectivity of our approach, we calculated the odds of two unrelated genomic events taking place by chance at the same genomic position, in both the human and mouse genomes. For this purpose, we applied the same above procedures on human and mouse SNPs located on chromosome X. This resulted in only 1.8 conserved SNPs (normalized to a database size of 443,366 SNPs in human and 453,726 in mouse) retrieved by the same BLAST parameters. Thus, we measured the signal-to-noise ratio at the editing set to be at least 32 (Figure 1D). Taken together, these results indicate that our set of sites is both robust and accurate.

**Table 1:** Mammalian evolutionarily conserved sites

	**chr**	**Position**	**Strand**	**Gene**	**Region**	**ref_id:nucleotide cange:aa change**
1	chr1	160302244	-	COPA	CDS	NM_001098398:c.A490G:p.I164V
2	chr11	105804694	+	GRIA4	CDS	NM_000829:c.A2293G:p.R765G
3	chr11	105815132	+	GRIA4	intron	
4	chr11	105816106	+	GRIA4	intron	
5	chr11	105816129	+	GRIA4	intron	
6	chr11	105816145	+	GRIA4	intron	
7	chr11	105816160	+	GRIA4	intron	
8	chr12	5021742	+	KCNA1	CDS	NM_000217:c.A1198G:p.I400V
9	chr13	46090371	+	COG3	CDS	NM_031431:c.A1903G:p.I635V
10	chr14	26917530	-	NOVA1	CDS	NM_006489:c.A1087G:p.S363G
11	chr14	101506074	+	mir376C	microRNA	
12	chr17	43045220	-	C1QL1	CDS	NM_006688:c.A197G:p.Q66R
13	chr19	47152854	-	DACT3	CDS	NM_145056:c.A775G:p.R259G
14	chr2	20450819	-	PUM2	3′UTR	
15	chr2	21233202	-	APOB	CDS	NM_000384:c.C6538G:p.Q2180stop
16	chr2	210835613	+	UNC80	CDS	NM_032504:c.A7990G:p.S2664G
17	chr20	36147533	-	BLCAP	CDS	NM_001167821:c.A44G:p.K15R
18	chr20	36147563	-	BLCAP	CDS	NM_001167821:c.A14G:p.Q5R
19	chr20	36147572	-	BLCAP	CDS	NM_001167821:c.A5G:p.Y2C
20	chr20	36148080	-	BLCAP	intron	
21	chr20	52104918	+	TSHZ2	3′UTR	
22	chr21	30953750	-	GRIK1	CDS	NM_175611:c.A1862G:p.Q621R
23	chr21	34922801	+	SON	CDS	NM_032195:c.A1264G:p.T422A
24	chr21	34923319	+	SON	CDS	NM_032195:c.A1782G:p.L594L
25	chr21	46595620	+	ADARB1	intron	
26	chr3	53820892	+	CACNA1D	CDS	NM_001128839:c.A4791G:p.I1597M
27	chr3	58141801	+	FLNB	CDS	NM_001164319:c.A6815G:p.Q2272R
28	chr3	62423807	-	CADPS	CDS	NM_183393:c.A3512G:p.E1171G
29	chr4	57976234	-	IGFBP7	CDS	NM_001253835:c.A284G:p.K95R
30	chr4	57976286	-	IGFBP7	CDS	NM_001253835:c.A232G:p.R78G
31	chr4	158257875	+	GRIA2	CDS	NM_000826:c.A1820G:p.Q607R
32	chr4	158257879	+	GRIA2	CDS	
33	chr4	158258136	+	GRIA2	intron	
34	chr4	158258137	+	GRIA2	intron	
35	chr4	158281294	+	GRIA2	CDS	NM_000826:c.A2290G:p.R764G
36	chr5	156736808	+	CYFIP2	CDS	NM_001037332:c.A958G:p.K320E
37	chr6	34100903	-	GRM4	CDS	NM_000841:c.A371G:p.Q124R
38	chr6	44120349	+	TMEM63B	CDS	NM_018426:c.A1856G:p.Q619R
39	chr6	102337689	+	GRIK2	CDS	NM_001166247:c.A1699G:p.I567V
40	chr6	102337702	+	GRIK2	CDS	NM_001166247:c.A1712G:p.Y571C
41	chr6	102372589	+	GRIK2	CDS	NM_001166247:c.A1862G:p.Q621R
42	chr6	102372630	+	GRIK2	intron	
43	chr6	102374616	+	GRIK2	intron	
44	chr6	102374643	+	GRIK2	intron	
45	chr6	150093334	+	PCMT1	intron	
46	chr8	103841636	-	AZIN1	CDS	NM_148174:c.A1099G:p.S367G
47	chr8	103841637	-	AZIN1	CDS	NM_148174:c.A1098G:p.E366E
48	chr9	97847739	+	mir23B	microRNA	
49	chrX	114082682	+	HTR2C	CDS	NM_000868:c.A466G:p.I156V
50	chrX	114082684	+	HTR2C	CDS	NM_000868:c.A468G:p.I156M
51	chrX	114082689	+	HTR2C	CDS	NM_000868:c.A473G:p.N158S
52	chrX	114082694	+	HTR2C	CDS	NM_000868:c.A478G:p.I160V
53	chrX	122598962	+	GRIA3	CDS	NM_000828:c.A2323G:p.R775G
54	chrX	122598998	+	GRIA3	intron	
55	chrX	135111055	+	SLC9A6	intron	
56	chrX	135111070	+	SLC9A6	intron	
57	chrX	151358319	-	GABRA3	CDS	NM_000808:c.A1026G:p.I342M
58	chrX	153579737	-	FLNA	intron	
59	chrX	153579950	-	FLNA	CDS	NM_001456:c.A6998G:p.Q2333R

List of conserved editing sites. Coordinates are based on the genome version GRCh37/hg19. For each site, the table includes the following information: chromosome, genomic coordinate, strand, gene name, genomic compartment, RefSeq id (if available), editing transformation, and coordinate related to the Refseq ID, and amino acid change (for the same Refseq ID).

### More data do not guarantee a greater number of ESS

**Figure 2:**
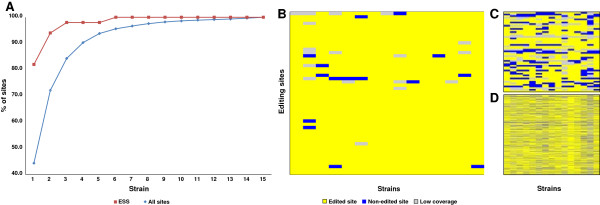
**The size of the ESS is almost independent of data accumulation. (A)** An accumulation curve of editing sites per strain (data derived from Danecek et al., whole brain samples). Strain datasets are sorted in ascending order of editing site amount (that is, the first strain contains the least number of editing sites, the second is the strain with the least additional editing sites, and so on). This result shows that addition of data does not lead to the addition of more conserved sites. **(B-D)** Visualization of sites per strain, ESS **(B)**, random sites selected from all sites in the same proportion as the ESS **(C)**, and all other sites **(D)**. Editing signal is colored in yellow; sites with no data, that have, fewer than three reads are colored in gray, and sites with no evidence for editing are colored in blue. The heat-maps demonstrate a strong editing signal for conserved sites over all mice strains in contrast to the non-conserved sites.

As sequencing data accumulated, the total number of identified editing sites increased as well. However, we found that the number of the evolutionarily selected editing sites did not increase when new sequencing data were added. Even though the set is rather small, its sensitivity (recall) and specificity rates seem to be strikingly high. The high recall rate was confirmed as the set contains all the conserved functional sites known so far. To measure the specificity of the ESS, we estimated the effect of accumulating a species-specific RNA editing dataset on the ESS size. In order to demonstrate that the size of the ESS does not dramatically change as the data accumulate, we found that even a small fraction of the available data is sufficient to retrieve most of the ESS. We used data from 15 mouse strains [4] to build a site accumulation curve (Figure 2A, Additional file 2: Figure S1). We found that for any random choice of two strains, we retrieved at least 94% of the sites (and only 72% of all other sites), and reached full saturation after including only six strains. Thus, the ESS is less sensitive to dataset enlargement compared to the set of all other mouse sites.

There are two reasons for explaining the few cases in which the editing signal was not detected in a specific mouse strain. The first is the low read depth (low expression), which makes measurement of editing levels difficult. The second explanation is editing levels under the detection threshold (or no editing at all). There is clear distinction between the conserved and the non-conserved sites: while there were only a few cases of sufficient coverage with no signal for editing in the conserved sites, there were a higher proportion of sites with sufficient coverage but no editing signal for the non-conserved ones. Many of the latter are not reproduced across different samples [53]. Visualization of the editing signal per strain by heat-maps is shown in Figure 2B-D. Our findings suggest that the selected sites tend to be edited in almost all mouse strains, while the non-conserved sites showed a weaker tendency for such consistency.

### Editing profiles for ESS in an additional four mammals

We analyzed RNA-seq data to find editing levels for the matched position of the ESS in four additional mammals: rat, cow, the non-placental marsupial opossum, and the monotrem platypus [54,55]. They were chosen based on the quality of their genomes, their evolutionary distance, and the availability of the necessary RNA-seq data. Although we analyzed only limited RNA-seq data per organism, we observed strong evidence for editing in the matched ESS for these species (Additional file 1: Table S3). In the rat transcriptome, 93.7% (45 out of 48) of the sites with a minimal coverage’s (>5 reads) exhibit evidence for editing. Similarly, 100% (23 out of 23) of sites with such coverage in the cow are edited. As expected, the very evolutionary distant opossum and platypus exhibit somewhat less evidence of editing with approximately 67% (21 out of 31 and 16 out of 24, respectively) of covered sites. These results are very strong, since we used only one or two RNA-seq datasets per organism. For example, in a single RNA-seq, derived from human brain, only 83% of the sites were found to be edited (Additional file 1: Table S4). We failed to find evidence for matched editing at only four sites (2 intronic in Gria4, and 2 coding Dact3 and C1ql1) in any the tested organisms, mainly due to low coverage for those sites.

### Genomic location of ESS

**Figure 3:**
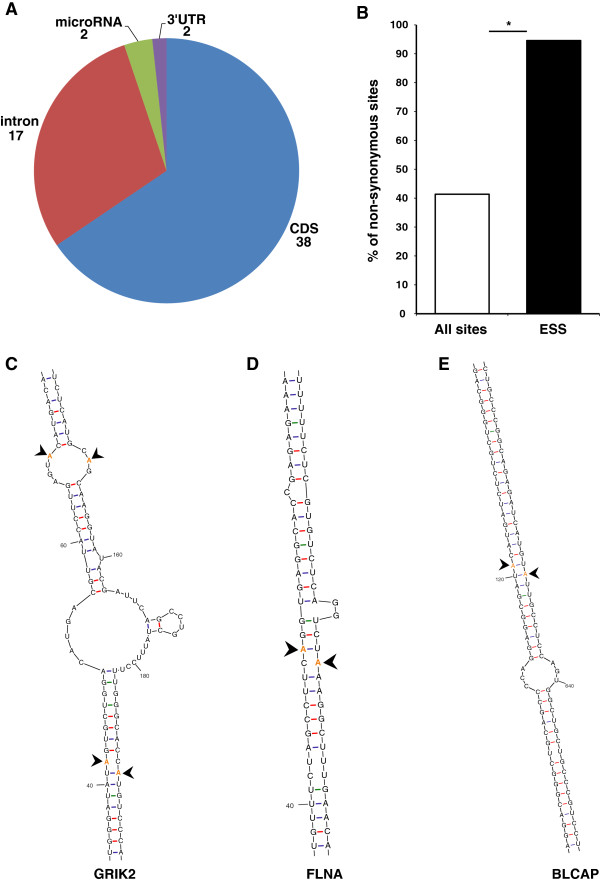
**Most of the ESS sites are located in a coding region or adjacent to such a site. (A)** Genomic location of evolutionarily conserved sites. **(B)** Frequency of non-synonymous editing alterations in exonic sites for both groups demonstrates enrichment of sites that cause amino acid change in the ESS compared to the control (all other sites, *P* <2 × 10^-11^ calculated by Fisher’s exact test). **(C-E)** Secondary structure shows spatial proximity of coding and intron sites of GRIK2 **(C)**, FLNA **(D)** and BLCAP **(E)** genes; editing sites are highlighted in orange and marked by an arrow.

Our results indicate that although both human and mouse transcriptomes contain a large number of editing sites, only a small group of the sites are shared as far back as the common ancestor of rodents and primates. These sites have different genomic characteristics *versus* the non-conserved sites. As expected, most of the sites in the ESS are located in coding regions (37 sites, 64%), (Figure 3A) and an absolute majority of these sites (35 sites, 94%) lead to non-synonymous mutations, which is a driving force in adaptive evolution (Figure 3B). The only two synonymous sites are located proximately to other non-synonymous site in the SON and AZIN genes, and their editing may be only a residual activity of ADAR near the main site.

We also found a relatively high number of sites located in introns (17 sites, 29.3%). In contrast to exons that have a clear potential for evolutionary benefit, such as amino acid changes, introns are considered as fitness-neutral in nature, and the reason they are evolutionarily conserved might look enigmatic. One probable explanation for the editing sites found in introns, is that the intronic sites are located in exon complementary sequence (ECS), a genomic region needed for dsRNA structure formation, which is required for ADAR binding [56]. Indeed, we found that 13 of the 17 intronic sites (76.4%) are located in genes that have recoding events as well (while only 11.1% for the control non-conserved intronic sites). Furthermore, by using secondary RNA structure prediction software (mfold) [57] for those sites (Figure 3C-E), we were able to confirm that 11 of 13 sites are located in complementary regions of other conserved editing sites (Additional file 2: Figure S2). In one interesting case, we found that the dsRNA structure was formed between two inverted introns. This structure contains a cluster of five proximate sites located in the GRIA4 gene. The extreme conservation of the two inverted introns and the five sites they harbor suggests that at least one of them has a functional role (Additional file 2: Figure S2A). Identification of the intronic ECS of a site is intrinsically important to study the function of the editing event in the coding region. As demonstrated in the past for several editing sites [10,44,58,59], a transgenic mouse with the unedited mRNA transcript can be generated by removal of the intronic ECS sequence. The identification of new ECS will allow functional studies of the corresponding editing sites.

Three additional sites were found in non-coding regions. Two sites are located in miRNAs (although short non-coding RNA sequences are usually depleted in RNA-seq libraries). The first one, previously published [18], is located within the seed region of mir-376c (Additional file 2: Figure S3). This editing event can alter the predicted targets of this miRNA. The second, an un-annotated conserved site, is located in mir-27b (Additional file 2: Figure S3). This miRNA is known to regulate many key processes such as lipid metabolism, inflammation and angiogenesis [60,61]. The third site is located within the 3′ UTR of the TSHZ2 gene. The site is located within the highly conserved 200 nt region, and we found a cluster of another four sites 150 nt upstream to this site in the mouse dataset. Notably, 11 of the sites are annotated as SNPs in dbSNP. Such erroneous annotation has been demonstrated in many of the previously identified editing sites [62,63], as sequences undergoing A-to-I RNA editing could be incorrectly identified as an A/G SNP. Former methods to discover SNPs used RNA sequences as well, and thus may be subject to this error. Indeed, the annotation of such SNP at dbSNP indicates that this SNP was detected by analysis performed on a cDNA library.

### Motif sequence

**Figure 4:**
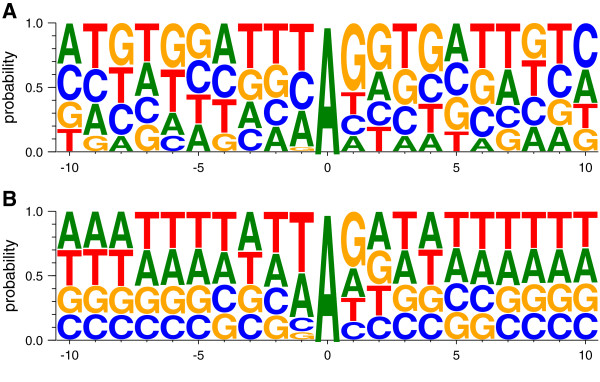
**Neighbor preferences for ESS and all sites.** Nucleotide frequency for ESS **(A)**, and all non-*Alu* sites **(B)**. Both signatures are in agreement with the ADAR motif.

Previous studies indicated that ADARs have a sequence preference for G depletion on the upstream nucleotide to the editing site and have excess of G at its 3′ base. We compared the nucleotide frequency for both the ESS and a control set (all human non-*Alu* sites). Although both sets adhered to the previously published neighbor preferences [64,65] (Figure 4), the motif signal of the ESS was stronger than the control, probably due to a purifying selection effect. Similar results were found in the mouse set, as well. This result supports the idea that the large un-conserved human and mouse sets do contain mostly genuine editing sites, but only very few are evolutionarily selected.

### Conserved sites have higher editing and expression levels

**Figure 5:**
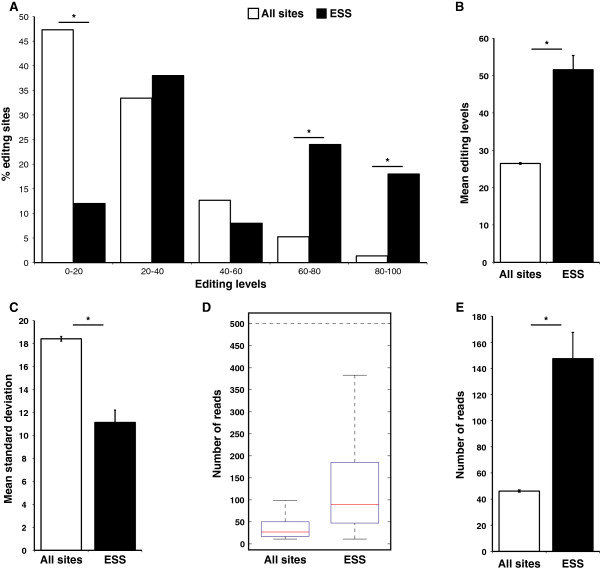
**ESS exhibit significantly higher and more consistent editing levels and higher expression levels compared to all other sites. (A)** Distribution of editing levels for ESS (black) and all other sites (white) (*P <10^-6^, Fisher’s exact test). **(B)** Mean editing levels for ESS *versus* all other sites (*P <7 × 10^-22^, Student’s two-tailed t-test). **(C)** Mean standard deviation for ESS and control (*P <4.6 × 10^-8^, two-tailed Student’s t-test). **(D, E)** ESS exhibits higher expression levels, as demonstrated by box plot **(D)** and by mean expression levels **(E)** (*P <10^-28^, two-tailed Student’s t-test).

We calculated the distribution of editing levels for the ESS and for all of the other previously published [4] sites in mouse (Figure 5A). Editing levels of a site are defined by the following ratio: (the number of G reads)/(the total number of A and G reads) of the base calling at the specific editing position. We found an over-representation of sites with high editing levels in the ESS compared to all other sites. Comparing the average editing levels revealed two-fold higher editing levels in the ESS (Figure 5B). High editing levels imply that the edited transcript is functional. In addition, we examined expression levels, by counting the coverage of each site. Here, again, we found that ESS sites have significantly higher expression levels than the remaining sites (Figure 5C-D), which also implies their significant function.

Further support for the idea that the ESS are subjected to strong selective pressure and are highly regulated is the consistency [66] of the editing levels between the different mouse strains. While the standard deviation between editing levels across 15 mice strains was rather high at the non-conserved sites, the same calculation revealed a striking conservation of editing levels in the ESS (11.1 for ESS, 18.4 for other sites, *P* = 4.6 × 10^-8^; two-tailed Student’s t-test, Figure 5E). Furthermore, we found a very high degree of similarity (correlation r = 0.93) of editing levels between two separate, recently published editing datasets [4,50] from mouse brain. In addition, we found consistent editing levels between human and mouse although millions of years of evolution separate them (r = 0.55 for all the ESS, and r = 0.77 for coding sites only, Additional file 2: Figure S4 and Additional file 1: Table S1). These findings indicate the ESS were selected due to the function they provide to the edited transcript. In addition, we made an editing level map of all the conserved sites in 16 different human tissues, by analyzing the available human-body-map RNA-seqs (Additional file 1: Table S4). As expected, we found that the most highly edited tissue is the brain. This result is consistent with the function of the conserved edited genes and with the fact that the majority of the mouse RNA-seq data originated from the brain. Although in general the editing levels of most of the sites are low [28] and therefore have a limited effect on the final protein product, the editing levels of the conserved sets are rather high and are similar for both human and mouse (average of 51.5% and 51.4%, respectively). For 27 sites in human and 25 in mouse (19 in both) the edited version is dominant and has more transcripts than the genomic encoded ones (>50% editing levels). A list of editing levels in human and mouse is provided in Additional file 1: Table S1.

The most commonly edited genes are BLCAP and IGFBP7, which are edited in all 16 tissues, while COG3, TSHZ2, SON, COPA, PUM2, AZIN1, and FLNA genes are found to be edited in at least 10 tissues. All the sites are located in coding sequences or in the 3′ UTR. This finding supports the hypothesis that coding sites are the main functional targets of ADARs, while intronic editing events probably represent residual ADAR activity. By counting the total number of edited reads for each site, assuming that the number of reads is correlated to expression levels, we found that the K/R site in IGFBP7 is the primary ‘consumer’ of ADARs in the human brain.

### Functional impact of editing

RNA editing increases the diversity of the genomic outcome in a specific locus by creating A or G variants. Specific gene families such as ion channels can utilize this capacity for rapid diversity by recoding genomic information. Indeed, we found that our set is enriched with GO terms that are related to neuron-specific functions, such as synaptic transmission, ion transport, and genes involved in neuroactive ligand-receptor interaction pathway (Additional file 1: Table S5). The editing of genes that encode proteins involved in neuronal excitability such as ion channels and transporters creates plasticity that can be used in response to environmental changes [67]. Comparing the ESS genes and proteins with other human genes and proteins revealed an unexpected result. The edited transcripts tend to be significantly longer than the average length of unedited human transcripts (5,674 bp in the ESS, 2,750 bp for human average transcripts, similar results for mouse). Similarly, the same trend was observed in the protein length (1,098 aa in the ESS and 328 for all human proteins). We have no apparent explanation for this phenomenon other than that longer genes have greater chances of being co-transcriptionally edited. Interestingly, the main C-to-U editing target of APOBEC1, the apoB transcript, encodes for an extremely long protein (4,563AA). This site is located in one of the largest encoding exons of the human genome (>7,500 bp), further strengthening the connection between editing and long transcripts.

### Editing tends to preserve an ancient version of the genome

**Figure 6:**
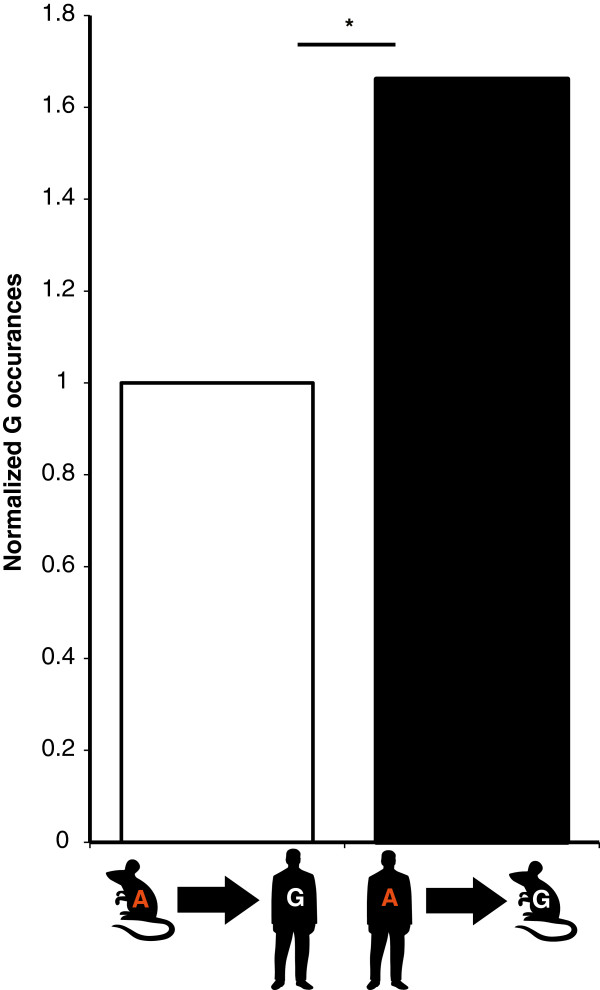
**A-to-I editing as a mechanism for the reversion of G-to-A evolution.** All mouse editing sites were converted to human genome coordinates. G-to-A ratio was calculated and fixed as 1 (left). All human editing sites were converted to mouse genome coordinates; G-to-A ratio was calculated and normalized (right), exhibiting 1.66-fold enrichment compared to the mouse-to-human conversion. (*P = 10^-7^, Fisher’s exact test).

An additional benefit of RNA editing is the ability to mitigate evolutionary changes, thereby maintaining evolutionary equilibrium. All editing positions have, by definition, a genomic adenosine. This adenosine is not always found in the matched position in genomes of other organisms. In the instances where there was no adenosine at the matched position, we found a majority of cases with ‘G’ hardwired at the counterpart genomic positions. Interestingly, when comparing the prevalence of genomic G in human and mouse sites, we found an asymmetric phenomenon. While 26% of all human editing sites with matching position (total of 12,937 sites) harbor a G in the mouse genome, only 18% (out of 1,083 such sites) of mouse editing sites harbor G in the human genome (Figure 6, *P* = 2.1 × 10^-7^, Fisher’s exact test). As a control, we applied the same procedure to adjacent but non-edited As. Here, no significant trend was found, and a similar percent (19%) of the human and mouse sites have a G at the corresponding position, suggesting this result is specific for edited adenosine. These results suggest that in the majority of cases, editing serves as a mechanism to compensate for a loss of phenotype caused by G-to-A evolution. This versatile reversion may occur in only part of the transcripts in parallel to the non-edited version and in a tissue-specific manner. Thus, editing allows the functional co-existence of two independently evolved genome versions. Furthermore, our results suggest that in addition to the ESS, there are additional functional editing sites in humans that have a G in the mouse genome (Additional file 1: Table S6), and therefore, were not included in this screen.

### Editing is involved in exonization of the LINE retroelement

**Figure 7:**
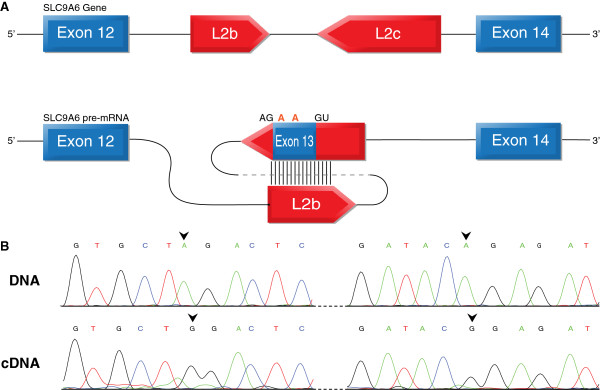
**Editing and exonization in the SLC9A6 gene. (A)** Schematic illustration of exons 12 to 14 of the SLC9A6 gene. Exons are depicted as blue boxes; the LINE inverted repeats are depicted as red boxes. Sense and antisense LINEs are expected to create a dsRNA secondary structure, thereby allowing RNA editing. The two editing sites are indicated in orange, revealing an R/G amino acid change. **(B)** Validation of editing by Sanger sequencing, genomic DNA (upper panel) and cDNA (lower panel) from the same individual; editing sites are marked by arrows.

Although most of the conserved editing sites are located in conserved regions of a protein (or in the ECS region), we found at least one editing site which seems to be involved in exonization of a retroelement (Figure 7A). In this unique case (SLC9A6), we found two editing sites located in a newly emerged exon derived from an L2 repeat. This is one of the first documented cases of preserved LINE exonization throughout mammalian evolution [68]. Since the complementary LINE that enables the editing is also conserved, a rare event by itself, we can assume that all three rare events (exonization of LINE, conservation of two LINEs, and two conserved editing events) are related, suggesting that editing was a driving force for the exonization and conservation of this element. The close proximity of editing to the splicing site provides additional supporting evidence for the involvement of editing in this exonization. The alternatively spliced exon is located in the SLC9A6 gene which has been implicated in several disorders causing mental retardation [69]. The gene product is the NHE6 protein, a hydrogen sodium exchanger. This channel controls the pH inside endosomes, which is important for the proper function of these compartments. Moreover, this ion exchanger was found to regulate clathrin dependent endocytosis of transferrin. The insertion of the alternative exon enlarges the protein by 30 amino acids, starting at position 507. The exon inclusion creates a longer C-terminal cytoplasmic tail. The editing sites convert the arginine (basic polar, positive side chain) at positions 511 and 514 to the non-polar and neutral glycine (R511G and R514G). Validation of the presence of this exon and editing sites is shown in Figure 7B. We believe this case is a unique example in which editing contributes to creation of new functional units.

### Insights from the vertebrate set

We also analyzed RNA-seq data to identify RNA editing candidates in zebrafish (see Methods). We used the same method to find conserved sites between human and mouse and zebrafish, which is one of the most evolutionarily distant vertebrate relatives of human for which genome and transcriptome data are available. We found 17 ESS sites that are also conserved in zebrafish (Additional file 1: Table S7), and most of them (10) are located in glutamate receptors. This enrichment is consistent with the high repertoire of glutamate receptors found in the zebrafish genome. The genomic sequence of the ESS sites is highly conserved across a diverse set of distant mammals (mouse, dog, and opossum) and includes most of the intronic ECS regions as well, suggesting that editing also takes place in these mammals too. Moreover, most of the sequences (45 sites, 76%) are also highly conserved in non-mammalian vertebrates (chicken and zebrafish, see Additional file 1: Table S8).

### Non-conserved editing sites

The large editing datasets we used for human and mouse were compiled from transcriptome wide analysis, which is subject to a high false positive rate, as previously described [20]. But, while the false positive rate in Alu regions is relatively low, the contamination of artifacts in non-repetitive regions is very high; in some cases the noise levels in these regions are even higher than the A-to-G signal [20]. Knowing that the non-conserved set is noisy, allows us to describe it only in general terms. Unlike the conserved one, this set exhibits inconsistent editing events across samples (Figure 2). In addition, the sites in this set are usually located in fitness neutral regions, as >90% of sites are located in introns and a majority of the coding sites lead to synonymous substitution (Figure 3B). Moreover, sites in this set exhibit low and variable editing levels (Figure 5B-C), and relatively low expression levels, as well (Figure 5D-E). Additional evidence for the stochastic nature of editing at these sites in this set, is that only 13.9% of the human specific sites exhibit an editing signal in any of the 16 tissues represented in the human body map, while a majority of the ESS (64.3%) show clear signal for editing in this dataset (Additional file 1: Tables S4 and S9).

A list of non-synonymous non-conserved sites is provided in Additional file 1: Table S10.

## Discussion

In this study, we defined, for the first time, the mammalian RNA editing set. Our results indicate that the conserved mammalian set of editing sites is extremely small and has distinct characteristics compared to the non-conserved sites. The conserved set has a unique genomic regional distribution, and has higher and more consistent editing levels, and higher expression levels than the non-conserved sites. Our results support the claim that only dozens of conserved functional editing sites exist, and negate the assumption that next-generation sequencing technologies will lead to the discovery of many additional novel conserved sites. One of those few targets, the SLC9A6 gene, demonstrates the evolution of an RNA editing site. This event is a result of dsRNA structure formation by the insertion of two inverted repeats, and the fixation of the locus across all mammalian lineages, probably due to the selective advantage provided by this sequence. A newly evolved site might be harmful, beneficial, or neutral. A harmful site will be eliminated quickly over the course of evolution. Conversely, a beneficial site will be conserved across evolution, and a neutral site will be eliminated at a slower evolutionary pace. It makes sense to assume that most of the lineage-specific sites are of neutral evolutionary fitness. Accordingly, it would be interesting to further investigate what advantage is provided by SLC9A6 editing.

Thirteen intronic sites were found to be located in a complementary sequence required for formation of dsRNA structure of another conserved editing site. When looking for the possible complementary regions for all sites, we were able to find the same strong evolutionary sequence conservation of more than 70 bp (out of 81) identity in 45 of the sites (See Additional file 1: Table S11). The remarkable conservation of two adjacent genomic regions for each site indicates that editing is the driving force for this conservation.

RNA editing can preserve a mutated genome version at the RNA molecule. This mechanism is beneficial, as it enables the simultaneous use of two evolved versions of an mRNA (the edited and the non-edited forms), at very low evolutionary cost [70]. Interestingly, we see clear evidence of editing to reverse evolutionary mutations, as opposed to editing being merely a ‘stepping stone’ for A-to-G genomic mutation.

Our studies revealed a comprehensive set of mammalian conserved sites. Yet, it is still possible that additional sites do exist and can be found if more samples from additional tissues (as most of the mouse data are derived from brain and liver) or genomes with higher coverage are used, or if less stringent criteria are used for conservation. However, our results suggest that adding more data or using relaxed parameters will not add many additional sites that are as highly edited and highly expressed. Therefore, we believe that this set is close to being inclusive, and if such additional sites do exist they are probably few in number.

## Conclusions

We carried out the first systematic screening for conserved mammalian RNA editing. Although we explored comprehensive editing sets in human and mouse, we found that there are only a few RNA editing sites that are conserved between these species. Our results demonstrate that although additional RNA-seq data can lead to the identification of novel editing targets, they are unlikely to add many additional conserved sites. We found that the mammalian conserved set of editing sites is limited and has distinguishing characteristics that set these sites apart from others. The conserved sites have a unique genomic distribution and tend to be located in regions with evolutionary impact. Most of the conserved edited genes are related to neural-specific functions; yet, we found an editing signal in a wide variety of tissues. In addition, we found editing to be involved in the creation of a new functional unit by exonization of a repetitive element in the SLC9A6 gene. Finally, we showed that editing tends to preserve ancient genome versions and allows the co-existence of two evolved phenotypes.

## Methods

### Creation of candidate RNA editing datasets

The human RNA editing dataset was created by merging published datasets. These datasets were created by seeking RNA-DNA differences using RNA-seq analysis. The first dataset that we used contained all editing candidates identified by Ramaswami et al. [28], who applied their previously published [20] pipeline to map RNA-seq reads. Their dataset includes canonical A-to-I sites and non-canonical sites, validated or not, in repetitive and non-repetitive regions, resulting in about 1 million sites, most of them identified in the brain. The second and third [24] datasets were created by identifying RNA editing candidates using an analysis of transcriptome and genome sequencing data of a lymphoblastoid cell line from the same individual, revealing 147,029 and 446,670 sites, respectively. The fourth dataset was downloaded from DARNED [47]. All datasets were merged into single dataset containing 1,432,744 unique editing sites.

The mouse RNA editing dataset was created by merging five datasets; the main dataset was created by Danecek et al., who screened for RNA-DNA differences, using whole brain RNA-seq analysis in 15 different mouse strains [4].

The second dataset published by Lagarrigue et al., investigated for RNA-DNA differences in liver and adipose tissues revealing 63 and 188 editing candidates, respectively [49]. The third dataset consisted of 176 A to I editing candidates, expressed in cerebral cortex [50]. The fourth dataset included 24 validated mouse A to I sites [48]. The fifth dataset was downloaded from DARNED [47].

### Zebrafish (ZF) editing dataset

This dataset was created by analysis of RNA-seqs (SRA Accession numbers: SRR1028002, SRR1028003, SRR1028004). Fastq files were aligned to the ZF genome (Zv9/DanRer7) using tophat, command: tophat -r 530 index fastq1,fastq1_replication fastq2,fastq2_replication fastq3,fastq3_replication. We then realigned the fastq files to the zebrafish reference genome, and added the splice junction file, achieved from the first run, as input.

Command: tophat -r 530 -j splice_junctions_file index fastq1,fastq1_replication fastq2,fastq2_replication fastq3,fastq3_replication.

Mpileup was then used to find RNA to reference-genome differences. Only sites with more than five edited reads and editing levels higher than 0.01 were taken into account.

### cDNA SNPs dataset

Human cDNA SNPs were downloaded from dbSNP137 (All SNPs table), using the UCSC table browser [52]. We made a filter for SNPs that were annotated as 1 nucleotide length cDNA SNPs only, revealing 79,152 cDNA SNPs.

### Aligning editing sites flanking regions between species

The sequences surrounding each editing site were downloaded using the UCSC table browser (versions: human-GRCh37/hg19, mouse-NCBI37/mm9). We used several sequence sizes and compared them by calculating signal-to-noise ratio, using SNP conservation as a control (Additional file 2: Figure S5). We chose to use a sequence of 40 nucleotides both upstream and downstream to the editing site, resulting in an 81 nucleotide sequence for each editing site. Nucleotide-nucleotide blast [51] (version: Nucleotide-Nucleotide BLAST 2.2.25+) was used to perform interspecies sequence alignment. Typical command: blastn -query *organism1_dadaset.fa* -strand ‘both’ -db *organism2_blastdb* -out *out_file* -task ‘dc-megablast’.

We then defined a 70 identity threshold to ensure either high identity levels or long alignment length. Additionally, we restricted all hits to have an exact match on the edited nucleotide (the edited nucleotides from each species are matched and aligned).

Mouse editing and expression levels were obtained from a previously published study [4]. For each site we used the mean of its editing levels among all strains as published. Expression levels were calculated by reads count, using RNA-seq data from the strain with the highest reads depth (NOD_ShiLtJ).

### Finding editing levels in human body map

We aligned the Human-body-map dataset to the human genome (hg19), using Bowtie aligner [71] with liberal parameters that allow mismatch detection (−n 3, -l 20, -k 20, -e 140 -best). Following alignment, we collected all mismatches between the above reads to the reference genome. Mismatches in read positions with quality Phred score <30 were discarded. Editing levels was measured as # of G reads/# of A + G reads.

### Editing levels in rat, cow, opossum, and platypus

Each RNA-seq was aligned to the matched reference genome (rat-rn4, cow-BosTau7, opossum-momDom5, and platypus-ornAna1) using STAR aligner with default parameters. Mismatches were screened using the same procedure as with human body map. Accession numbers for RNAseq: cow- SRR594491, rat- SRR594419, opossum- SRR306744, SRR306746, platypus- SRR306727, SRR306729. Human body map- GSE7905.

Sequence logos were generated for 10 nt upstream and downstream to the editing sites using WebLogo. [72].

RNA secondary structures were predicted using mfold [57].

Codon changes were calculates using ANNOVAR [73].

## Abbreviations

ADAR: Adeonsine deaminase acting on RNA; BLAST: Basic local alignment tool; DARNED: Database of RNA editing; ECS: Exon Complementary Sequence; ESS: Evolutionary selected set; GLUR2: Glutamate receptor2; GO: Gene ontology; LINE: Long interspersed nuclear element; miRNA: microRNA; SNP: Single nucleotide polymorphism; UTR: Untranslated region.

## Competing interests

The authors declare that they have no competing interests.

## Authors’ contributions

YP performed the research, analyzed the data, and wrote the manuscript. HYC participated in the design of the study and writing. EYL conceived the study, helped to analyze the data, and to write the manuscript. All authors read and approved the final version of the manuscript.

## Supplementary Material

Additional file 1: Table S1 Additional information on the ESS, including human and mouse coordinates, year of publication, if they were found using next generation sequencing data analysis and molecular type of SNPs found in this position. **Table S2.** Additional sites that were found to be conserved by UCSC liftOver. **Table S3.** Editing profiles for the ESS in rat, cow, opossum, and platypus. For each organism we provide the matched nucleotide, number of A + G reads, and editing levels. **Table S4.** Editing levels among 16 Human body map tissues. **Table S5.** Enriched GO terms in the ESS. **Table S6.** Human editing sites which harbor ‘G’ in mouse genome. **Table S7.** ESS sites that were found to be edited in zebrafish; coordinates are in Zv9/DanRer7 genome version. **Table S8.** Sequence conservation among mouse, dog, opossum, chicken, and zebrafish. **Table S9.** Editing levels among 16 Human body map tissues for human specific sites (deleted regions in mouse). **Table S10.** List of all non-conserved non-synonymous sites. **Table S11.** List of predicted complementary region for each site in the ESS. Click here for file

Additional file 2: Figure S1 Incidence of editing sites per strain. The prevalence of editing sites was measured for the ESS (conserved sites) and all other sites (*P* value = 7.24 × 10-10, Student’s t-test). **Figure S2.** Spatial proximity of conserved sites. The secondary structure shows spatial proximity of the conserved sites of **(A)** gria3, and **(B)** five intronic sites in the gria4 gene. Editing sites are depicted in orange and marked by an arrow. **Figure S3.** Conserved editing sites in microRNAs. Editing sites in pre-mir **(A)**. The editing site is located in the seed region of mir376c. **(B)** Editing site within mir27b. Editing sites are highlighted in orange and marked by an arrow. **Figure S4.** Editing levels are conserved between human and mouse. RNA editing levels were measured in both human and mouse brains. We found positive correlation between editing levels in both species by calculating Pearson’s correlation coefficient (R = 0.55). **Figure S5.** Signal-to-noise ratios. Signal-to-noise was measured by the ratio of editing hits to normalized SNPs hits. Both were calculated using the pipeline as described in the paper. We used 40 nt, 80 nt, and 100 nt blast alignment length and the UCSC liftover. Click here for file
