# Supplementary material for: Mammalian conserved ADAR targets comprise only a small fragment of the human editosome
Source: Genome Biol. 2014 Jan 7;15(1):R5. doi: 10.1186/gb-2014-15-1-r5 (PMC4053846; doi:10.1186/gb-2014-15-1-r5)
Supplement: Additional file 2: Figure S1 — Incidence of editing sites per strain. The prevalence of editing sites was measured for the ESS (conserved sites) and all other sites (P value = 7.24 × 10^-10^, Student’s t-test). Figure S2. Spatial proximity of conserved sites. The secondary structure shows spatial proximity of the conserved sites of (A) gria3, and (B) five intronic sites in the gria4 gene. Editing sites are depicted in orange and marked by an arrow. Figure S3. Conserved editing sites in microRNAs. Editing sites in pre-mir (A). The editing site is located in the seed region of mir376c. (B) Editing site within mir27b. Editing sites are highlighted in orange and marked by an arrow. Figure S4. Editing levels are conserved between human and mouse. RNA editing levels were measured in both human and mouse brains. We found positive correlation between editing levels in both species by calculating Pearson’s correlation coefficient (R = 0.55). Figure S5. Signal-to-noise ratios. Signal-to-noise was measured by the ratio of editing hits to normalized SNPs hits. Both were calculated using the pipeline as described in the paper. We used 40 nt, 80 nt, and 100 nt blast alignment length and the UCSC liftover. [file gb-2014-15-1-r5-S2.docx]

**Mammalian conserved ADAR targets comprise only a small fragment of the human editosome**

Yishay Pinto, Haim Y. Cohen and Erez Y. Levanon

Mina and Everard Goodman Faculty of Life Sciences, Bar-Ilan University, Ramat Gan, Israel

**Additional file figures**

Contents

[Additional file Figure S1. 2](#_Toc363040235)

[Additional file Figure S2 3](#_Toc363040236)

[Additional file Figure S3. 4](#_Toc363040237)

[Additional file Figure S4. 5](#_Toc363040238)

[Additional file Figure S5. 6](#_Toc363040239)

# Additional file Figure S1.

**Additional file Figure S1**. Incidence of editing sites per strain. The prevalence of editing sites was measured for the ESS (conserved sites) and all other sites (p value = 7.24X10^-10^, Student’s t-test).


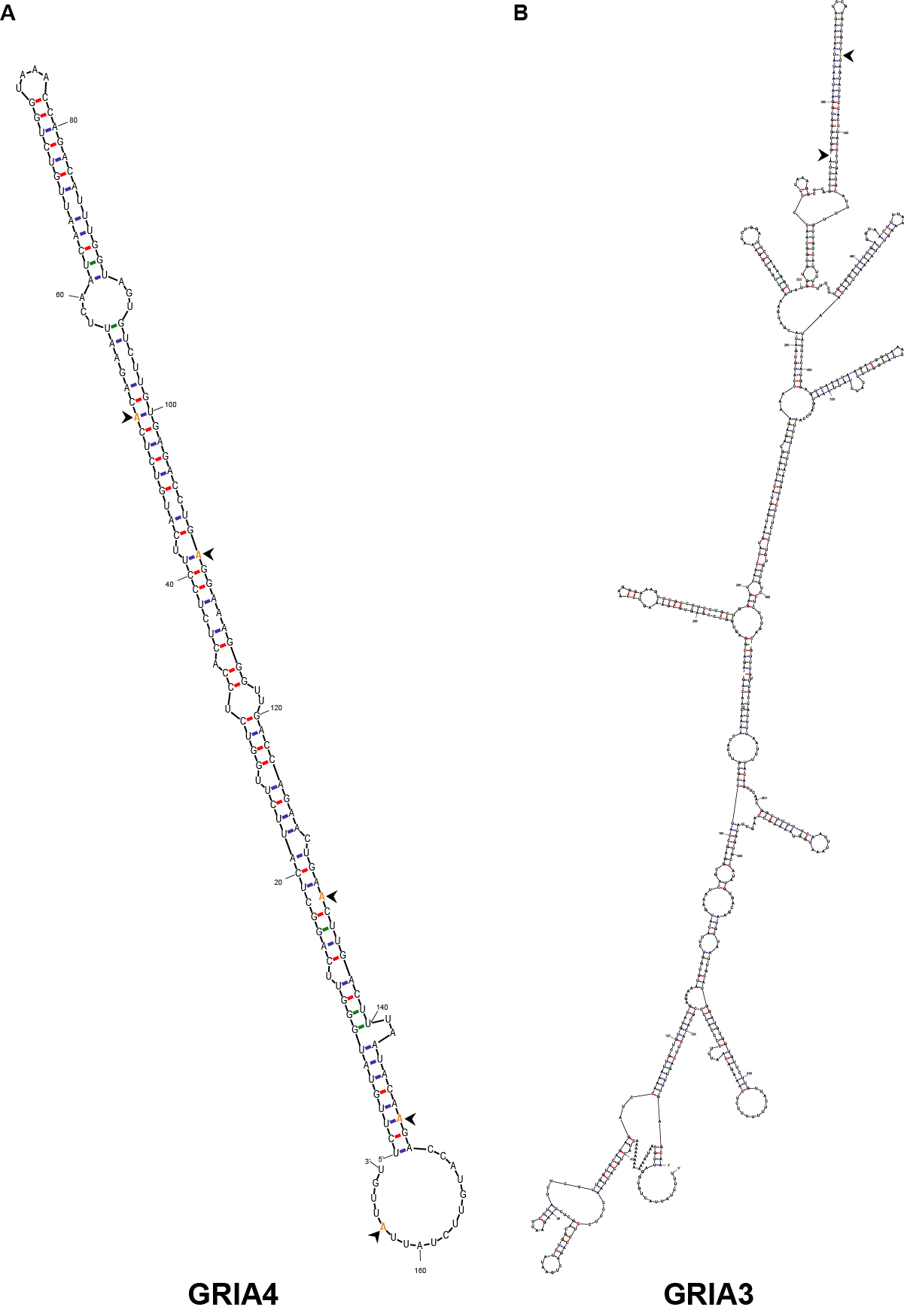
Additional file Figure S2**.**

**Additional file Figure S2. Spatial proximity of conserved sites.** The secondary structure shows spatial proximity of the conserved sites of (A) gria3, and (B) five intronic sites in the gria4 gene. Editing sites are depicted in orange and marked by an arrow.

# Additional file Figure S3.


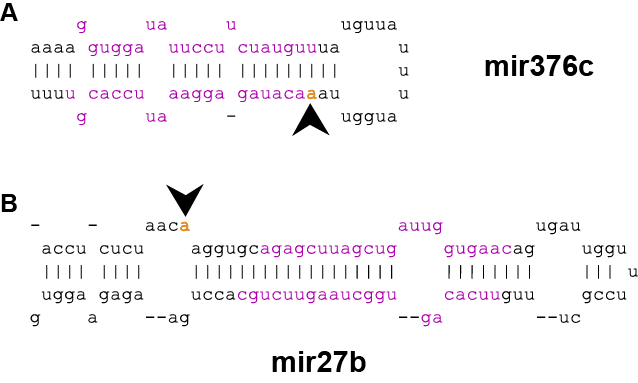


**Additional file Figure S3.** **Conserved editing sites in microRNAs.** Editing sites in pre-mir(A). The editing site is located in the seed region of mir376c. (B) Editing site within mir27b. Editing sites are highlighted in orange and marked by an arrow.

# Additional file Figure S4.

**
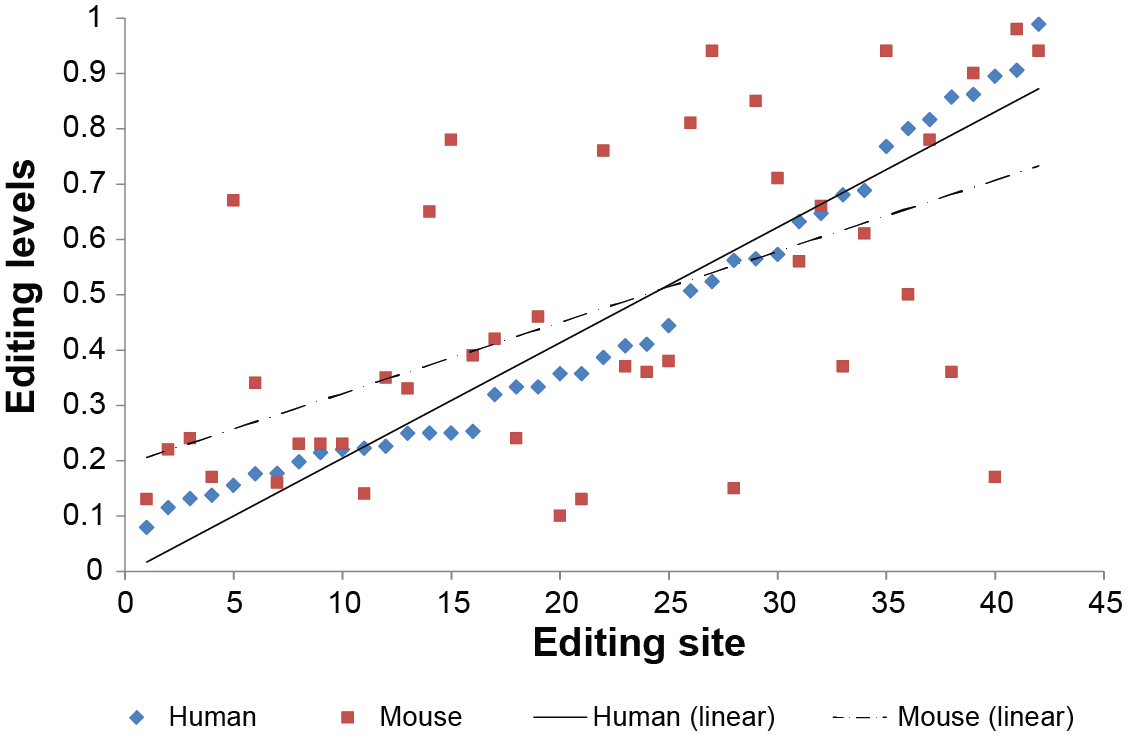
**

**Additional file Figure S4.** **Editing levels are conserved between human and mouse.** RNA editing levels were measured in both human and mouse brains. We found positive correlation between editing levels in both species by calculating Pearson's correlation coefficient (R=0.55).

# Additional file Figure S5.

**
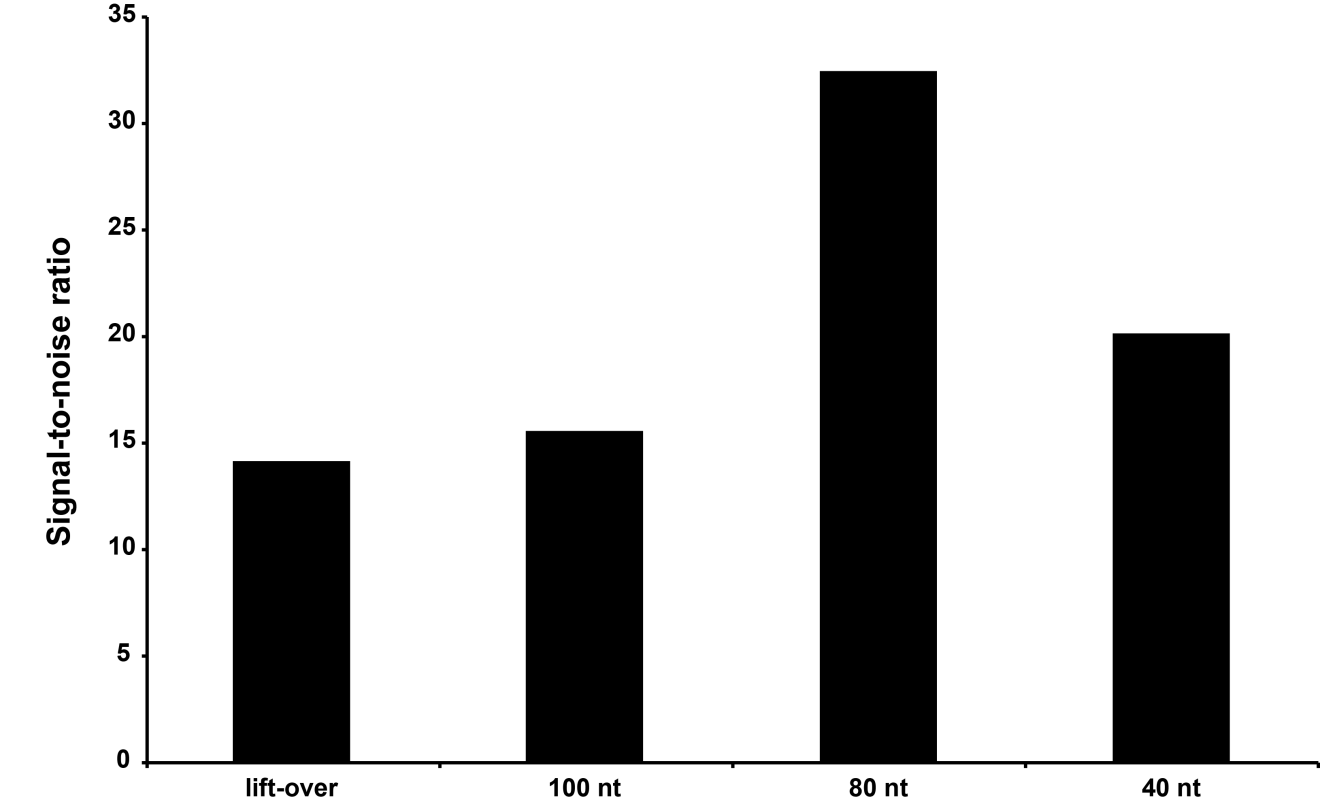
**

**Additional file Figure S5.** **Signal-to-noise ratios.** Signal-to-noise was measured by the ratio of editing hits to normalized SNPs hits. Both were calculated using the pipeline as described in the paper. We used 40nt, 80nt and 100nt blast alignment length and the UCSC liftover.
